# Anticancer Drugs Induced Severe Adverse Cutaneous Drug Reactions: An Updated Review on the Risks Associated with Anticancer Targeted Therapy or Immunotherapies

**DOI:** 10.1155/2018/5376476

**Published:** 2018-01-17

**Authors:** Chau Yee Ng, Chun-Bing Chen, Ming-Ying Wu, Jennifer Wu, Chih-Hsun Yang, Rosaline Chung-Yee Hui, Ya-Ching Chang, Chun-Wei Lu

**Affiliations:** ^1^Department of Dermatology, College of Medicine, Chang Gung Memorial Hospital, Keelung, Linkou, Taipei, Taiwan; ^2^Drug Hypersensitivity Clinical and Research Center, Chang Gung Memorial Hospital, Taipei, Taiwan; ^3^School of Medicine, College of Medicine, Chang Gung University, Taoyuan, Taiwan; ^4^Graduate Institute of Clinical Medical Sciences, Chang Gung University, Taoyuan, Taiwan

## Abstract

Cutaneous adverse drug reactions are commonly seen in patients with anticancer drug treatment. Anticancer drugs, including chemotherapy, target therapy, and recent immunotherapy causing skin reactions ranging from mild skin rash to life-threatening severe cutaneous adverse reactions (SCARs), such as Stevens-Johnson syndrome (SJS) and toxic epidermal necrosis (TEN) with increase morbidity and mortality while they are receiving cancer treatments, have been proposed to be a result of direct skin toxicity or drug hypersensitivity reactions (these are proposed mechanism, not definite). Differentiating SCARs from other more commonly seen reactions with a better outcome help prevent discontinuation of therapy and inappropriate use of systemic immunosuppressants for presumable allergic reactions, of which will affect the clinical outcome. In this article, we have reviewed published articles from 1950 to August 2017 for SJS/TEN associated with anticancer drugs, including chemotherapy, targeted therapy, and immunotherapy. We aimed to provide an overview of SJS/TEN associated with anticancer drugs to increase clinician recognition and accelerate future studies on the pathomechanism and managements.

## 1. Introduction

The advancement in cancer detection and development of anticancer drug therapy has led to increased incidence of cutaneous adverse reactions following anticancer drug therapy. Conventional chemotherapy and targeted or immunotherapy that are thought to be well tolerated and may cause various cutaneous adverse reactions ranging from nonlife-threatening skin toxicities such as paronychia, acneiform eruption, and alopecia to life-threatening severe cutaneous adverse reactions (SCARs) such as Stevens-Johnson syndrome (SJS) and toxic epidermal necrolysis (TEN). These drug eruptions are thought to be immunologically mediated reactions that are termed type B adverse reaction [1]. However, the pathomechanism of SCARs reactions in anticancer drugs including chemotherapy, targeted therapy, and immunotherapy is poorly understood and the literatures were still limited.

SJS/TEN are a spectrum of fatal mucocutaneous adverse reactions characterized by rapidly progressing purpuric atypical target-like rashes with blisters, cutaneous sloughing, and mucosal involvement. SJS and TEN are differentiated by the degree of skin detachment: SJS involves less than 10% body surface area skin detachment, TEN more than 30%, while SJS/TEN overlap involves body surface area of 10–30% [1, 2]. Despite their rare occurrence, the overall mortality was generally high in accordance with the body surface involve, ranging from 10% for SJS to approximately 50% for TEN, and can cause irreversible sequelae to the eyes, skin, and lungs [2–5]. Hence, increased recognition and improved management are of paramount importance, especially at early stages. Furthermore, in clinical practice, the conjectural association of anticancer drugs with SCAR event may lead to alterations in therapy, affects clinical outcome, and may cause physician and patient distress. This review aimed to provide an overview of the current evidence of anticancer drug-related SCARs to assist clinicians in early recognition and management.

To synthesize current literature, relevant English literatures were identified through searches of PubMed, EMBASE, Web of Science, SCOPUS, and OVID from 1950 to August 2017 using the terms Stevens-Johnson syndrome, toxic epidermal necrolysis, cancer drug therapy, and target therapy drugs. We did not constrain our research on publication types but limited the search only in indexed, peer-reviewed journals so as to ensure quality publications. Primary case reports, case series, reports from clinical trials, or as part of postmarketing surveillance were included. Histopathologic diagnosis of SJS/TEN was not required for the inclusion criteria. Clinical course, type of anticancer drugs, and mortality were analyzed and summarized according to the respective anticancer drug classifications of chemotherapy [6–54] (Table 1), targeted therapy [55–80] (Table 2), and immunotherapy [81–87] (Table 3). Cases with multiple concomitant medications used during the same period of time and/or with questionable diagnosis were excluded.

## 2. Chemotherapy

Chemotherapy is the most widely used anticancer drug in oncology field. The administration of chemotherapy may lead to many cutaneous findings, ranging from allergic reactions to infectious complications caused by disrupted immunity. From the search of peer-review articles, a total of 60 reports of SJS/TEN associated with 23 chemotherapeutic anticancer drugs were identified [6–54] (Table 1). The most common drugs to cause chemotherapy-induced SJS/TEN are lenalidomide (*n* = 14; SJS = 12, SJS/TEN = 1, and TEN = 1), methotrexate (*n* = 5; SJS = 2 and TEN = 3), docetaxel (*n* = 4; SJS = 3 and TEN = 1), and thalidomide (*n* = 5; SJS = 1 and TEN = 4). Most patients were exposed to drugs either concomitantly or within 8 weeks of the anticancer agent. Although there were a few cases with exceedingly short duration of onset with questionable diagnosis [28, 31], the report descriptions and causality indicators (course of treatment, duration and timing between exposure and event, blood levels, etc.) were not consistently reported in these articles. Some articles enclose pictures that are not very suggestive of SJS/TEN but of an alternative diagnosis, including erythema multiforme, GVHD, and toxic erythema of chemotherapy. For instance, methotrexate-induced epidermal necrosis is a distinct entity that closely mimics SJS/TEN but exhibits distinct clinicopathological features from SJS/TEN [88]. Many of the reported articles did not obtain skin biopsy for pathology examination and hence, it is difficult to draw to a definitive diagnosis of SJS/TEN. Another clinical mimic of SJS/TEN associated with chemotherapy is toxic erythema of chemotherapy (TEC), characterized by painful erythematous eruptions with edema and/or blisters which involves the acral part, intertriginous areas, pressure points, and less often ears, knees, and elbows [89, 90]. TEC is a toxic phenomenon with minimal inflammatory infiltrates despite the dramatic clinical appearance, hence studies have hypothesized that the erythema is secondary to keratinocyte damage with release of cytokines leading to vasodilation [90, 91]. Most cases involve the use of either antimetabolites or alkylating agents that interferes RNA or DNA synthesis, including methotrexate, cytarabine, 5-fluorouracil, and mercaptopurine. By contrast, SJS/TEN is an immune-driven type 4 allergic reaction, where cytotoxic T lymphocytes and natural killer cells are activated. Clinical recognition and differentiation of SJS/TEN from toxic erythema are of importance because it helps prevent the inappropriate use of systemic immunosuppressants for presumed allergic reactions, precludes subsequent dosing, and affects the patient's clinical outcome.

## 3. Targeted Anticancer Therapy

From the literature review, a roster of 42 reports of SJS (*n* = 23), SJS/TEN (*n* = 4), or TEN (*n* = 15), associated with 12 targeted anticancer drugs, were identified, including EGFR inhibitors (afatinib, cetuximab, erlotinib, gefitinib, panitumumab, and vandetanib), MKI (imatinib, regorafenib, and sorafenib), recombinant IL-2 (aldesleukin), proteasome (bortezomib), anti-CD20 (rituximab), anti-CD30 (brentuximab vedotin), and BRAF inhibitor (vemurafenib) (Table 2). The most common drugs to cause SJS/TEN reported are imatinib (*n* = 11), EGFR inhibitors (*n* = 10), and vemurafenib (*n* = 7). The response of cancer control is hard to analyze because it was not fully mentioned in the reports. All cases were treated with immunosuppressant, including steroid, IVIG, and there was one TEN case with promising outcome after etanercept (anti-TNF *α*) treatment. In these reports, nine patients underwent drug rechallenge test with recurrences, confirming the notoriety of exposed targeted anticancer drugs [67–72, 74, 80].

### 3.1. EGFR Inhibitors

EGFR inhibitors are approved as the drug for the treatment of non-small cell lung, colorectal, breast, pancreatic, head, and neck cancers with EGFR mutations [92]. The incidence of EGFR inhibitor-induced cutaneous adverse drug reactions (cADRs) is high (36%–80%) [93], of which most were papulopustular eruptions, xerosis, paronychia, mucositis, and photosensitivity [94]. In this article, we have identified 13 cases of SJS/TEN induced by EGFR inhibitors. Though rare, SJS/TEN should be distinguished from EGFR inhibitor-related mucositis, particularly when the patient present with constitutional symptoms and widespread atypical target spots with blisters that extend beyond mucosa to the skin. Cross-reactivity between EGFR inhibitors was reported. It is hypothesized that the pathomechanism of SJS/TEN associated with EGFR inhibitors could be caused by to the irreversible inhibition of EGFR, of which hinders epidermal differentiation and reepithelialization and causing extensive erosions [95].

### 3.2. KIT and BCR-ABL Inhibitors

Imatinib, a tyrosine kinase inhibitor, is the standard treatment in chronic myeloid leukemia and gastrointestinal stromal tumors (GIST) [96, 97]. In this article, imatinib accounts one of the most common causative targeted anticancer drug to induce SJS, with a roster of 12 cases. This must be differentiated from other more commonly seen cutaneous adverse effects of imatinib, maculopapular rashes, and facial edema [98], of which has a better prognosis and dose-dependent pharmacologic effect rather than hypersensitivity reaction [99]. For maculopapular rash/facial edema associated with imatinib, temporary discontinuation or dose reduction may be applied if the patient's cancer is susceptible to the drug. By contrast, reintroducing the culprit drug with a dose reduction is usually not suggested [100, 101].

### 3.3. Multikinase Inhibitors

Multikinase inhibitors (sunitinib, sorafenib, pazopanib, and vandetanib) are small molecule inhibitors of the tyrosine kinase of the VEGF, and also differential binding capacities to other tyrosine kinases, including PDGFR, EGFR, KIT, RET, FLT-3, CSF-1R, and RAF [102]. They were approved for treatment of patients with renal cell cancer, gastrointestinal stromal tumors, and hepatocellular cancer. These drugs can cause hand-foot skin reaction, hair change, maculopapular eruptions, stomatitis, genital erosions, and bleeding [103, 104], especially in patients using sorafenib. These more common cutaneous toxicities are thought to be caused by direct VEGF inhibition, which result in vessel regression, and impact on vascular repair capacities [74]. Other research has also shown that Fas/FasL interaction mediates keratinocyte death in sunitinib-induced HFSR [75]. Recently, one recent study identified SLC22A20 (OAT6) as an uptake carrier of sorafenib and subsequently sorafenib enters the keratinocyte through OAT6 and then inhibits mitogen-activated protein kinase MAP3K7 (TAK1) leading to cytotoxicity and keratinocyte injury [76]. Interestingly, erythema multiforme, a spectrum of delayed type hypersensitivity, induced by sorafenib was around 19–25% in Japanese population, which is much higher than the Caucassian population [105]. This could imply a possible genetic role in the pathogenesis of adverse drug reactions. The different incidence of cutaneous adverse reactions among different ethnicities need to be further investigated.

### 3.4. BRAF Inhibitors

Vemurafenib is a selective inhibitor of BRAF-kinase approved for the treatment of metastatic melanoma with BRAF mutation. Skin toxicity, such as photosensitivity and maculopapular eruptions, and secondary skin malignancy (keratoacanthoma and squamous cell carcinoma) were estimated to affect more than 90% of patients [106, 107]. One vemurafenib-TEN underwent a lymphocyte transformation test (LTT) assay to confirm the causality of vemurafenib and also show positive cross-reactivity for dabrafenib [108]. On the contrary, another case reported a successful switch from vemurafenib-induced cutaneous adverse reactions to dabrafenib [109]. Furthermore, cross-reactivity was also found between vemurafenib and sulfonamide antibiotics—sulfamethoxazole—based on LTT reports. These data suggested that there might be clinical cross-reactivity between BRAF inhibitors and sulfonamides. Predisposing factors to sulfonamide-related adverse cutaneous drug reactions could be implied in the pathomechanism studies of vemurafenib-associated SJS/TEN [108].

### 3.5. mTOR Inhibitors

Mammalian target of rapamycin (mTOR) inhibitors, such as sirolimus, everolimus, and temsirolimus, are emerging drugs, increasingly applied in oncology and in the prevention of rejection in patients receiving solid organ transplantation [110]. The most common cutaneous side effects are oral ulcers, acne-like eruptions, and morbilliform drug eruptions [111]. Oral ulcer is a very frequent (72%) adverse reaction and is often recurrent and chronic following everolimus treatment in 25% of patients. The adverse event was found to be dose dependent [112].

Severe drug eruptions of life-threatening lingual angioedema after initiation of everolimus in heart transplant recipients have also been reported in a case series. In these patients, lingual edema occurs predominantly within the first weeks after initiation of everolimus therapy and disappears without recurrences in majority patients after adequate symptomatic treatment [113].

There were otherwise no SCAR (SJS/TEN, DRESS) event being reported in the literature.

## 4. Immunotherapy

Immunotherapy is the latest breakthrough in anticancer drug development with immunomodulatory therapeutic antibodies, targeting inhibitory receptors expressed by T cell as CTLA-4 and PD-1. They are used to treat advance stage cancer with metastasis or unresectable tumor such as melanoma and lung cancer. In this section, older immunotherapy such as interleukin-2 was also included in Table 3. These therapeutic options are most widely used in advanced and late cancer stages. From literature reviews, we have identified one ipilimumab-SJS, two nivolumab-TEN, and four pembrolizumab-SJS. All of the patients were advanced melanoma patients, and the onset of epidermal necrolysis varies from 2.5 weeks to 3 months. In one case of pembrolizumab-associated SJS, concomitant phenytoin for epilepsy was used; hence, the exact culprit drug is hard to define. Two cases of pembrolizumab-SJS were being reported by Saw et al. [114]. Interestingly, there was a striking demarcation of epidermal detachment along the radiotherapy field aside from typical mucocutaneous findings of SJS. Such findings, although rarely, have also been reported in previous traditional culprit drugs and targeted therapy. A total of 3 cases were found with interleukin-2 immune therapy with 2 fatalities [81, 82, 87]. One of the authors suggested that IL-2 may increase patient's susceptibility to allergy of other medication [87]. An increased expression of PD-L1 in the epidermis by immunohistochemistry (IHC) was found, and they hypothesized that the use of anti-PD-1 therapy could provoke the expression of PD-L1 of keratinocytes and permit the activated CD8^+^ cytotoxic T cells to target keratinocytes leading to keratinocyte apoptosis [86]. PD-1 knockout mouse often exhibits symptoms related to adverse cutaneous reactions. It has been reported in a mouse model that PD-L1 expressed on keratinocytes presenting self-antigens regulates autoreactive CD8^+^ T cell activity and prevents the development of cutaneous autoimmune disease [115]. Goldinger et al. had demonstrated that the gene expression analysis of TEN-like lesional skin from anti-PD-1-treated patients revealed an upregulation of major inflammatory chemokines, such as CXCL9, CXCL10, and CXCL11, of cytotoxic mediators such as PRF1 and GZMB and proapoptotic FASLG and upregulation of PD-L1 [116]. These gene expression profiles resembling SJS/TEN suggest that PD-1/PD-L1 interaction is required to preserve epidermal integrity during inflammatory skin reactions. Interestingly, there was a case with preceding nivolumab treatment followed by vemurafenib who developed TEN [117]. The authors suggest that nivolumab predispose patients to drug hypersensitivity reactions through activation of CD8^+^ cells [84, 85].

In spite of being uncommon, SJS/TEN are severe life-threatening cutaneous diseases that should be concerned in patients treated with anticancer drugs. The typical presentation and diagnosis often require proper drug exposure documentation, photography, and skin biopsies. Currently, there are many different classifications and models with detail and validated diagnostic criteria to assist clinical diagnosis and can help predict patients' mortality [118, 119]. Standard reporting method is important for subsequent investigation and analysis of these rare events. In addition, diagnosis of culprit drug is often challenging, the drug notoriety scoring systems including ALDEN score, Naronjo score and in vitro test with lymphocyte transformation test (LTT) are useful tests for the diagnosis of drug hypersensitivity and cross-reactivity and helped to better understand these reactions [120, 121]. Current evidence on the pathomechanism of this complication was limited. Further research is warranted to elucidate the pathophysiology as well as help clinician coping with this notorious adverse event, advancing towards personalized medicine in oncology treatment.

## Figures and Tables

**Table 1:** Anticancer chemotherapy-related severe cutaneous adverse drug reactions from the English literature (year: 1950–2017).

Drug class	Drug	Pharmacology	References	Total (*n*)	Mortality	SJS	SJS/TEN	TEN
Alkylating agents	Treosulfan	Alkysufonates	[6]	1	1	0	0	1
	Chlorambucil	Mustard gas derivatives	[7, 8]	2	0	0	0	2
	Mechlorethamine (topical)	Nitrogen mustard	[9]	1	0	1	0	0
	Temozolomide	Hydrazines and triazines	[10]	1	0	0	1	0
	Procarbazine	Hydrazines and triazines	[11–13]	3	0	0	0	3

Plant alkaloids	Paclitaxel	Taxanes	[14]	1	0	1	0	0
	Docetaxel	Taxanes	[15–19]	5	2	3	0	2
	Etoposide	Podophyllotoxins	[20]	1	0	1	0	0

Anthracyclines	Doxorubicin		[21]	1	1	0	0	1

Antimetabolites	Methotrexate	Folic acid antagonists	[22–26]	5	2	2	0	3
	Cytarabine	Pyrimidine antagonist	[27, 28]	2	2	0	0	2
	Fludarabine	Adenosine deaminase inhibitor	[29]	1	1	1	0	0
	Gemcitabine	Pyrimidine antagonist	[30–32]	3	0	2	1	0
	Capecitabine	Pyrimidine antagonist	[33]	1	0	1	0	0
	Cladribine	Purine antagonist	[34, 35]	2	NA	1	0	1
	6-Mercaptopurine	Purine antagonist	[36]	1	NA	0	0	1
	TS-1 (tegafur-gimeracil-oteracil potassium)		[37, 38]	2	0	1	0	1
	Pemetrexed	Multitarget antifolate	[39, 40]	2	0	0	0	2

Antitumor antibiotics	Bleomycin		[41, 42]	2	1	0	0	2
	Peplomycin		[43]	1	0	1	0	0
	Mithramycin		[44, 45]	2	0	0	0	2

Miscellaneous	Lenalidomide		[46–48]	14	2	12	1	1
	Thalidomide		[49–53]	5	1	1	0	4
	Asparaginase		[54]	1	0	0	0	1
			Total	**60**	**13**	**28**	**3**	**29**

NA: not available.

**Table 2:** Anticancer targeted therapy-related severe cutaneous adverse drug reactions from the English literature (year: 1950–2017).

Drug class	Drug	Pharmacology	References	Total (*n*)	Mortality	SJS	SJS/TEN	TEN
EGFR inhibitor	Afatinib	Monoclonal antibody to EGFR	[55, 56]	2	0	2	0	0
	Cetuximab	Monoclonal antibody to EGFR	[57–59]	4	1	1	1	2
	Erlotinib	TKI specific to EGFR	[60]	1	0	1	0	0
	Gefitinib	TKI specific to EGFR	[61, 62]	2	1	0	0	2
	Panitumumab	Monoclonal antibody to EGFR	[122]	1	0	1	0	0
	Vandetanib	Less specific multikinase inhibitors	[63]	2	0	0	1	1

KIT and BCR-ABL inhibitors	Imatinib	KIT, BCR-ABL, PDGFR	[64–72]	11	1	11	0	0

Antiangiogenic agents	Sorafenib	Nonselective antiangiogenesis multikinase agents	[73–76]	3	0	2	0	1

Proteasome	Bortezomib		[77]	2	1	1	0	1

CD30	Brentuximab vedotin	CD30	[78]	2	0	1	0	1

CD20	Rituximab	Monoclonal antibody to CD20	[79]	5	2	2	2	1

BRAF inhibitors	Vemurafenib	A/B/C-Raf and B-Raf (V600E)	[80]	7	1	1	0	6
			Total	**42**	**7**	**23**	**4**	**15**

**Table 3:** Anticancer immune therapy-related adverse drug reactions from the English literature (year: 1950–2017).

Drug class	Drug	Pharmacology	References	Total	Mortality	SJS	SJS/TEN	TEN
Immunomodulators	Aldesleukin	Recombinant interleukin-2	[81, 82]	2	1	0	0	2
	Ipilimumab	CTLA-4 inhibitors	[83]	1	0	1	0	0
	Nivolumab	PD-1 inhibitors	[84, 85]	2	1	0	0	2
	Pembrolizumab	PD-1 inhibitors	[86, 114, 116]	4	0	4	0	0
	Denileukin	Recombinant interleukin-2 and diphtheria toxin	[87]		1	0	0	1
			Total	9	3	5	0	5
